# Plasmonic Pd Nanoparticle- and Plasmonic Pd Nanorod-Decorated BiVO_4_ Electrodes with Enhanced Photoelectrochemical Water Splitting Efficiency Across Visible-NIR Region

**DOI:** 10.1186/s11671-016-1492-8

**Published:** 2016-06-04

**Authors:** Weiwei Yang, Yunjie Xiong, Liangliang Zou, Zhiqing Zou, Dongdong Li, Qixi Mi, Yanshan Wang, Hui Yang

**Affiliations:** Shanghai Advanced Research Institute, Chinese Academy of Sciences, Shanghai, 201210 China; University of Chinese Academy of Sciences, Beijing, 100039 China; School of Physical Science and Technology, ShanghaiTech University, Shanghai, 201210 China

**Keywords:** Plasmonic Pd, BiVO_4_, Photoelectrochemical water splitting, Surface plasmon resonance

## Abstract

The photoelectrochemical (PEC) water splitting performance of BiVO_4_ is partially hindered by insufficient photoresponse in the spectral region with energy below the band gap. Here, we demonstrate that the PEC water splitting efficiency of BiVO_4_ electrodes can be effectively enhanced by decorating Pd nanoparticles (NPs) and nanorods (NRs). The results indicate that the Pd NPs and NRs with different surface plasmon resonance (SPR) features delivered an enhanced PEC water splitting performance in the visible and near-infrared (NIR) regions, respectively. Considering that there is barely no absorption overlap between Pd nanostructures and BiVO_4_ and the finite-difference time domain (FDTD) simulation indicating there are substantial energetic hot electrons in the vicinity of Pd nanostructures, the enhanced PEC performance of Pd NP-decorated BiVO_4_ and Pd NR-decorated BiVO_4_ could both benefit from the hot electron injection mechanism instead of the plasmon resonance energy transfer process. Moreover, a combination of Pd NPs and NRs decorated on the BiVO_4_ electrodes leads to a broad-band enhancement across visible-NIR region.

## Background

Solar hydrogen generation through photoelectrochemical (PEC) water splitting offers an efficient and sustainable solution to the global energy problem [1–5]. Recently, BiVO_4_ has emerged as a promising material for PEC water splitting due to its photoactivity in visible light region [6]. However, BiVO_4_ photoanodes suffer from rapid charge carrier recombination, slow water oxidation kinetics, and insufficient photoresponse in the spectral region with energy below the band gap of 2.4 eV [6], which limit its water splitting efficiency. Therefore, strategies such as doping [7–12], nanostructuring [13–18], and loading of oxygen evolution catalysts (OECs) [7, 9, 19, 20] have been adopted to improve the water splitting efficiency of BiVO_4_.

As reported, several dopants, such as W, Mo, and P, are reported to improve the PEC performance of BiVO_4_ [7–12]. Doped BiVO_4_ exhibits the improved carrier density, enhanced conductivity, or even increased hole diffusion length, thus resulting in enhanced PEC properties. Furthermore, the short diffusion length of photoexcited charge carriers is the main reason for the dominant electron-hole recombination in the bulk of BiVO_4_. To address this issue, the diffusion length for charge carriers can be shortened by nanostructuring, thereby reducing bulk recombination [13–18]. To increase the water oxidation kinetics, research efforts have been placed on the loading of the OECs on BiVO_4_ [7, 9, 19, 20]. Among these OECs, the Co-Pi and FeOOH catalysts can lead to a negative-shift of onset potentials of water oxidation and effectively enhance the magnitude of photocurrent [7, 9, 19]. However, through these strategies, the enhanced photoactivity of BiVO_4_ has only been achieved in the spectral region with energy above the band gap. To improve the PEC water splitting efficiency of BiVO_4_ in the spectral region with energy below the band gap or even in the near-infrared (NIR) region, whose energy accounts for 56.3 % of that of the solar spectrum [21], still remains a big challenge.

Recently, a new approach involving metal nanostructures in enhancing the photoactivity of TiO_2_ in the spectral region with energy below the band gap via plasmonic effect has received much attention [22–24]. Surface plasmon resonance (SPR) is an intrinsic property of metal nanostructures, in which the oscillation frequency is highly sensitive to their shape and size of the metal nanostructures as well as the dielectric constant of the surrounding environment [25–30]. The plasmonic metal nanostructures localize the optical energy by SPR and enhance the photoactivity of semiconductors through either near-field electromagnetic enhancement or hot electron injection [22–24]. For example, Hsu et al. reported that the performance of Au nanostructure-decorated TiO_2_ nanowires for PEC water splitting was enhanced across entire UV-visible region [23]. Although an enhancement in the PEC water splitting efficiency was also observed on BiVO_4_ electrodes with plasmonic metal nanostructures, such as Au and Ag [31–33], there are no reports that the photoactivity of BiVO_4_ can be improved in the spectral region with energy below the band gap or even in the NIR region by exploiting plasmonic metal nanostructures.

This work reports that the enhancement of PEC water splitting efficiency can be effectively extended into the visible-NIR region by the combination of Pd nanoparticles (NPs) and nanorods (NRs) with BiVO_4_ electrodes. The mechanisms of activity enhancement both in the visible and NIR regions have been discussed.

## Methods

### Preparation of BiVO_4_ Electrodes

The BiVO_4_ electrodes were prepared as previously reported procedure [19]. Solutions for electrodeposition were prepared by dissolving 10 mM Bi(NO_3_)_3_ (98 %, Alfa Aesar) in a solution of 35 mM VOSO_4_ (97 %, Sigma Aldrich) at pH < 0.5 with HNO_3_ (65 %, Acros Organics). Then 2 M CH_3_COONa (≥99.0 %, Alfa Aesar) was added, raising the pH to ∼5.1, which was then adjusted to pH 4.7 with a few drops of HNO_3_. Acetate serves to stabilize other insoluble Bi (III) ions at pH 4.7. This mildly acidic pH condition must be used because at pH lower than 2 where Bi(III) is soluble, no film can be formed while at pH higher than 5, V (IV) precipitates from solution. A three-electrode cell was used for electrodeposition, with an fluorine-doped tin oxide (FTO, 8 Ω/□, Hartford Glass Co.) coated glass substrate as working electrode, a Ag/AgCl (4 M KCl) as reference electrode and a platinum foil as counter electrode. A potentiostat (Sloartron SI 1287) was used for electrodeposition. Deposition of amorphous Bi–V–O films was carried out potentiostatically at 1.9 V vs Ag/AgCl (4 M KCl) for 5 min at 70 °C (ca. 2 mA cm^−2^). The as-deposited films were converted to crystalline BiVO_4_ and amorphous V_2_O_5_ by annealing at 500 °C for 1 h in air, and pure BiVO_4_ was achieved by dissolving the V_2_O_5_ in 1 M KOH under stirring for 20 min.

### Synthesis of Pd NPs and NRs

A simple but very efficient method that uses CO as reducing agent to synthesize Pd NPs and NRs has been developed and will be reported elsewhere. In brief, before the synthesis, 80 mM/L NaCl (≥99.0 %, Sigma Aldrich) and 40 mM/L PdCl_2_ (≥99.9 %, Sigma Aldrich) was dissolved in 50 mL CH_3_OH (≥99.9 %, Sigma Aldrich) to form Na_2_PdCl_4_ for preparation. Then, a flask was added 210-mg polyvinylpyrrolidone (K 30, average Mw 40,000, Sigma Aldrich) and 100 mL of 3.876 mM Na_2_PdCl_4_. To produce Pd NRs or NPs, 0.2- or 4.0-mL ultra-purity water (Millipore Milli-Q purification system, resistivity >18 MΩ cm) was added respectively. High-purity N_2_ with a flow rate of 200 mL/min was used for deaeration of the solution for 1 h under intense stirring. Then, high-purity CO with a flow rate of 200 mL/min was introduced to the flask under gentle stirring for 10 min. After that, a common balloon linked to the flask was blown up with CO to keep the reducing atmosphere for 4 h. During the whole synthesis process, the reaction system was keep at 30 °C.

### Preparation of Pd Nanostructure-Decorated BiVO_4_ Electrodes

Pd nanostructure-decorated BiVO_4_ electrodes were prepared using an electrophoretic deposition process. A field of 15 V cm^−1^ was applied to deposit Pd nanostructures using a FTO counter electrode held at positive potentials relative to the working electrode (BiVO_4_ electrode). For the preparation process of Pd NP-decorated BiVO_4_ (NP-BiVO_4_) and Pd NR-decorated BiVO_4_ (NR-BiVO_4_) electrodes, the depositing time are 3 and 1 min, respectively. To prepare Pd NP- and NR-decorated BiVO_4_ (NP-NR-BiVO_4_) electrodes, Pd NRs were firstly deposited onto the surface of BiVO_4_ for 30 s and then Pd NPs were deposited for 90 s. After this deposition process, Pd nanostructure-decorated BiVO_4_ electrodes were rinsed by distilled water and then dried under atmospheric environment for 2 h.

### Characterization

X-ray diffraction (XRD) was carried out using a Bruker AXS D8 Advance powder X-ray diffractometer with a Cu Kα (*λ* = 1.5418 Å) radiation source to confirm the purity and crystallinity of the prepared BiVO_4_ electrodes. UV-vis spectra were obtained using a Cary 5000 UV-vis-NIR spectrophotometer in diffuse reflectance mode. Scanning electron microscopy (SEM) images were collected with a field-emission SEM (Hitachi Situation-4800). Elemental compositions were determined by Energy Dispersive X-ray Spectroscopy (EDX) using the EDX detector on the Hitachi Situation-4800. Transmission electron microscopy (TEM) images were obtained with a JEOL 2100F at an accelerating voltage of 200 kV.

### Photoelectrochemical Measurements

All photoelectrochemical measurements were carried out in a 0.1-M potassium phosphate buffer (KPi) at pH = 7, using a three-electrode setup, with a Ag/AgCl (4 M KCl) as reference electrode and a platinum foil as counter electrode. Linear scanning voltammograms (LSVs) were obtained with a scan rate of 10 mV s^−1^. White-light photocurrent measurements were conducted under simulated AM 1.5G solar illumination with a Xe lamp coupled with a Newport Sol3A Class AAA solar simulator (94023A-SR3). The light intensity of the solar light simulator was calibrated to 100 mW cm^−2^ by the standard reference of a Newport 91150 silicon solar cell. Visible (400–800 nm, 98.7 mW cm^−2^) and NIR (800–2000 nm, 363 mW cm^−2^) light measurements were conducted with this Xe lamp couple with spectral filters. Monochromatic photocurrent measurements were conducted with this Xe lamp coupled with a set of monochromatic filters. The whole working electrode was illuminated through the back side of FTO glass. The potential vs Ag/AgCl (4 M KCl) can be converted against the reversible hydrogen electrode (RHE) using the following equation:

$$ E\left(\mathrm{v}\mathrm{s}\ \mathrm{R}\mathrm{H}\mathrm{E}\right) = E\left(\mathrm{v}\mathrm{s}\ \mathrm{A}\mathrm{g}/\mathrm{AgCl}\right) + {E}_{\mathrm{Ag}/\mathrm{AgCl}}\left(\mathrm{r}\mathrm{e}\mathrm{f}\right) + 0.0591\mathrm{p}\mathrm{H} $$

$$ \left({E}_{\mathrm{Ag}/\mathrm{AgCl}}\left(\mathrm{r}\mathrm{e}\mathrm{f}\right) = 0.1976\ \mathrm{V}\ \mathrm{v}\mathrm{s}\ \mathrm{R}\mathrm{H}\mathrm{E}\ \mathrm{at}\ 2{5}^{\mathrm{o}}\mathrm{C}\right) $$

## Results and Discussion

**Fig. 1:**
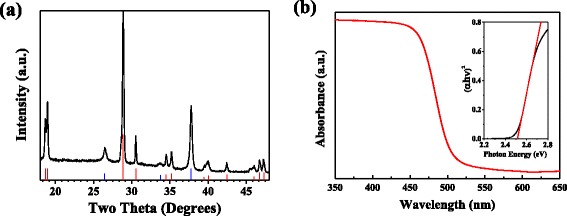
**a** XRD pattern of the BiVO_4_ film. The *vertical lines* indicate JCPDS diffraction peaks of FTO (*blue*) and monoclinic BiVO_4_ (*red*) and **b** UV-vis absorption spectrum of the BiVO_4_ film. *Inset* shows a Tauc plot of BiVO_4_ for direct bandgap

Figure 1a shows XRD pattern of the BiVO_4_ film, which can be matched to monoclinic BiVO_4_ (JCPDS No. 14–0688). In addition, UV-vis absorption spectrum of the BiVO_4_ film was collected and its bandgap was estimated to be 2.5 eV, which agrees well with values for the bandgap of BiVO_4_ reported in the literature [19] (Fig. 1b).

**Fig. 2:**
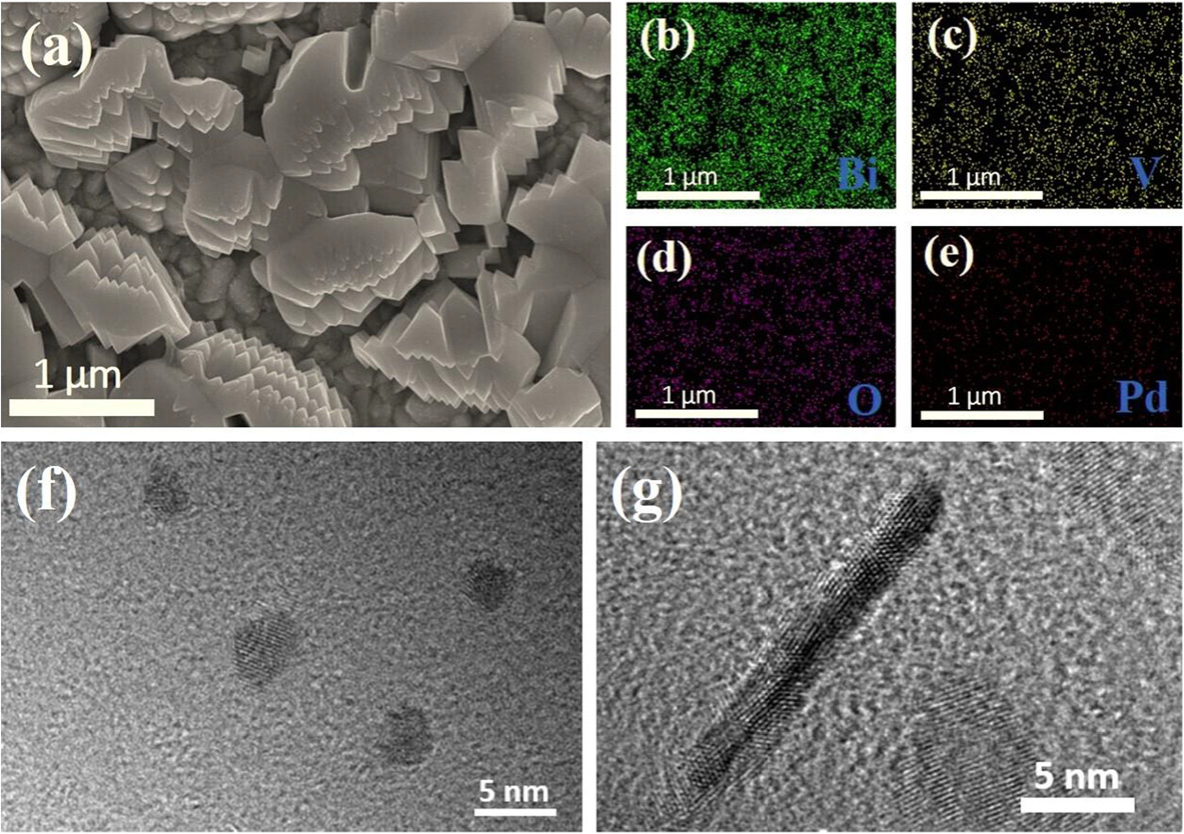
**a** Typical SEM image and **b**–**e** EDX maps of NP-NR-BiVO_4_ film for four elements. TEM images of Pd **f** NPs and **g** NRs

Figure 2 shows SEM image and EDX maps of as-prepared NP-NR-BiVO_4_ composite film and TEM images of Pd nanostructures. SEM image demonstrates that the BiVO_4_ film is composed of grains with no obvious feature. Although no Pd NPs and NRs can be observed in the SEM image due to their small size (Fig. 2f, g), EDX maps clearly show that Pd element presents a uniform distribution on the surface of BiVO_4_ film, with the Pd/Bi weight percentage being 2.17 %, indicating that Pd NPs and NRs are successfully decorated on the surface of BiVO_4_ film. EDX analyses for NP-BiVO_4_ and NR-BiVO_4_ electrodes show that the Pd/Bi weight percentages are 2.15 and 1.9 %, respectively.

**Fig. 3:**
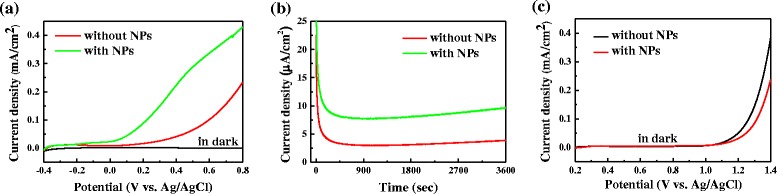
PEC performance of bare BiVO_4_ and NP-BiVO_4_ electrodes: **a** LSVs in the dark and under AM 1.5G illumination, **b** *I*–*t* curves under AM 1.5G illumination at 0.3 V, and **c** LSV in the potential range from 0.2 to 1.4 V under dark condition

Figure 3a shows linear scanning voltammograms (LSVs) of NP-BiVO_4_ and bare BiVO_4_ electrodes recorded in 1-M KPi solution in the dark and under AM 1.5G illumination (100 mW cm^−2^). The dark scans collected in the potential range between −0.4 and 0.8 V reveal a small background current of ∼10^−7^ A/cm^2^. Under AM 1.5G illumination, NP-BiVO_4_ and bare BiVO_4_ electrodes show a steady increase in photocurrent with applied potential. Importantly, NP-BiVO_4_ electrodes exhibited substantially larger photocurrent density than bare BiVO_4_ electrodes. The photocurrent density is ca. 0.336 mA cm^−2^ at 0.6 V on NP-BiVO_4_ electrode, which is about 3.3 times higher than that on bare BiVO_4_ electrode. Furthermore, chronoamperometric curves were collected during a period of 1 h at 0.3 V under AM 1.5G illumination. As shown in Fig. 3b, during such a relatively long period, the enhancement is still noticeable, suggesting that the photoactivity of BiVO_4_ electrodes can be enhanced under simulated solar light illumination by decoration of Pd NPs on the surface.

Figure 3c shows LSVs of bare BiVO_4_ and NP-BiVO_4_ electrodes collected in the potential range from 0.2 to 1.4 V under dark condition. There is a decrease in current on NP-BiVO_4_ electrode in the potential ranges between 1.1 and 1.4 V, which could rule out the catalytic effect of Pd NPs on water splitting. Such a current decrease could be ascribed to the fact that the coverage of Pd NPs on the surface of BiVO_4_ reduces the interfacial surface area between BiVO_4_ and the electrolyte, and thereby hindering the water oxidation.

**Fig. 4:**
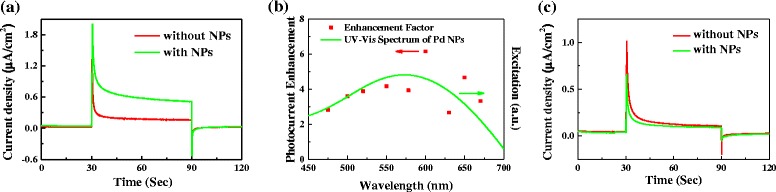
*I*–*t* curves at 0.4 V for bare BiVO_4_ and NP-BiVO_4_ electrodes under **a** monochromatic light (500 nm) and **c** NIR light illumination. **b** *Symbols*: photocurrent enhancement for NP-BiVO_4_ electrode as a function of excitation wavelength. *Solid curve*: absorption spectrum of the Pd NPs stock solution

To explore the possible reason for the enhanced PEC water splitting on the NP-BiVO_4_ electrodes, chronoamperometric curves were collected for bare BiVO_4_ and NP-BiVO_4_ electrodes under a set of monochromatic light illumination in the visible region. Figure 4a shows the photocurrent response of PEC water splitting on BiVO_4_ with and without the decoration of Pd NPs under 500 nm (60 mW cm^−2^) illumination, where the photocurrent on NP-BiVO_4_ is ca. 4 times of that on pristine BiVO_4_ electrode. The enhancement factor of photocurrent on the NP-BiVO_4_ electrode as a function of excitation wavelength was imposed on the absorption spectrum of synthesized Pd NPs stock solution, as shown in Fig. 4b. It can be seen that the enhancement factor strongly depends on the excitation wavelength and that it exhibits a similar trend with Pd NPs absorption spectral feature. Since the Pd absorption spectrum is a consequence of the SPR of Pd NPs, the result suggests that the Pd SPR could be responsible for the observed photocurrent enhancement [24]. Additionally, the photoresponses of bare BiVO_4_ and NP-BiVO_4_ electrodes under NIR illumination are obtained and shown in Fig. 4c. The photocurrent collected from NP-BiVO_4_ electrode slightly decreases in NIR region in comparison with BiVO_4_ electrode. This result is reasonable since the SPR feature of Pd NPs is located at 573 nm, which is far away from NIR region, and the SPR excitation of Pd NPs cannot occur under NIR illumination.

**Fig. 5:**
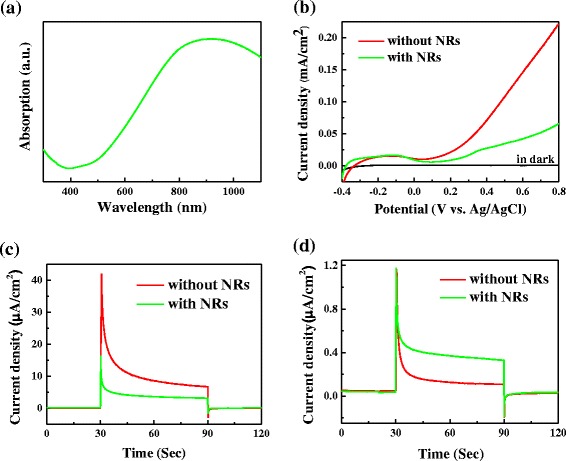
**a** UV-vis absorption spectrum of Pd NR stock solution. PEC performance of bare BiVO_4_ and NR-BiVO_4_ electrodes: **b** LSVs in the dark and under AM 1.5G illumination; *I*–*t* curves at 0.4 V with **c** visible light and **d** NIR light illumination

To extend the photoresponse to NIR region for the PEC water splitting, Pd NRs were decorated on the surface of BiVO_4_ because the SPR featured peak is located at ca. 900 nm (see Fig. 5a). Figure 5b–d displays the PEC properties of NR-BiVO_4_ and bare BiVO_4_ electrodes. As shown in Fig. 5b, the photocurrent density of NR-BiVO_4_ electrode under AM 1.5G illumination decreased dramatically compared to bare BiVO_4_ electrode. The photocurrent density on the NR-BiVO_4_ and BiVO_4_ electrodes at 0.6 V are 42 and 146 μA cm^−2^, respectively. To reveal the plasmonic effect of Pd NRs on the photoresponse of BiVO_4_ electrodes, photocurrent response for the NR-BiVO_4_ and bare BiVO_4_ electrodes was conducted under visible light and NIR light illumination, respectively. As shown in Fig. 5c, the photocurrent for the NR-BiVO_4_ electrode obtained under visible light illumination greatly decreased relative to bare BiVO_4_ electrode. Intriguingly, NR-BiVO_4_ electrode exhibited much enhanced photoactivity under NIR light illumination, as shown in Fig. 5d, indicating that the SPR excitation of Pd NRs is responsible for the enhanced photoactivity of NR-BiVO_4_ electrodes under NIR light illumination. To the best of our knowledge, this is the first report that the photoactivity of BiVO_4_ for water splitting can be enhanced in the NIR region by loading plasmonic metal nanostructures [31–33]. As for the photocurrent decrease under visible light and AM 1.5G illumination, it could be attributed to the fact that the presence of Pd NRs not only block the light absorption of BiVO_4_ but reduce the interfacial surface area between BiVO_4_ and the electrolyte, thus hindering the transfer of photoexcited holes into the interface between BiVO_4_ and the electrolyte for water oxidation.

**Fig. 6:**
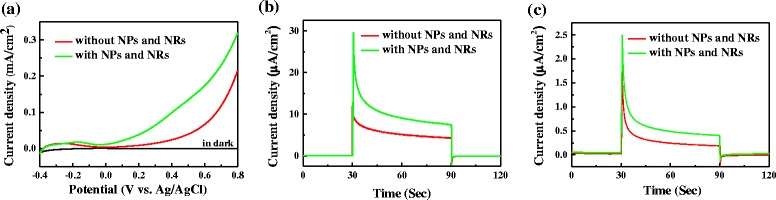
PEC performance of bare BiVO_4_ and NP-NR-BiVO_4_ electrodes: **a** LSVs in the dark and under AM 1.5G illumination; *I*–*t* curves collected at 0.4 V with **b** visible light and **c** NIR light illumination

Inspired by the facts that the NP-BiVO_4_ and NR-BiVO_4_ electrodes exhibit respectively enhanced PEC water splitting efficiency in the visible and NIR region, a combination of Pd NPs and Pd NRs with BiVO_4_ electrodes was fabricated by decorating a mixture of Pd NPs and NRs onto the surface of BiVO_4_ electrode. Figure 6 shows the PEC properties of NP-NR-BiVO_4_ and bare BiVO_4_ electrodes. As shown in Fig. 6a, NP-NR-BiVO_4_ electrode exhibited an enhanced photocurrent density in the potential range from 0.0 to 0.8 V. Importantly, chronoamperometric curves collected under visible and NIR light illumination as shown in Fig. 6b, c demonstrate that the NP-NR-BiVO_4_ electrode exhibited an enhanced PEC water splitting efficiency in both visible and NIR regions. Although the enhancement factors are not high enough, there must be an optimal loading for Pd NPs and NRs on the BiVO_4_ to achieve much better PEC performance. Nevertheless, this study is beyond the scope of the current work.

**Fig. 7:**
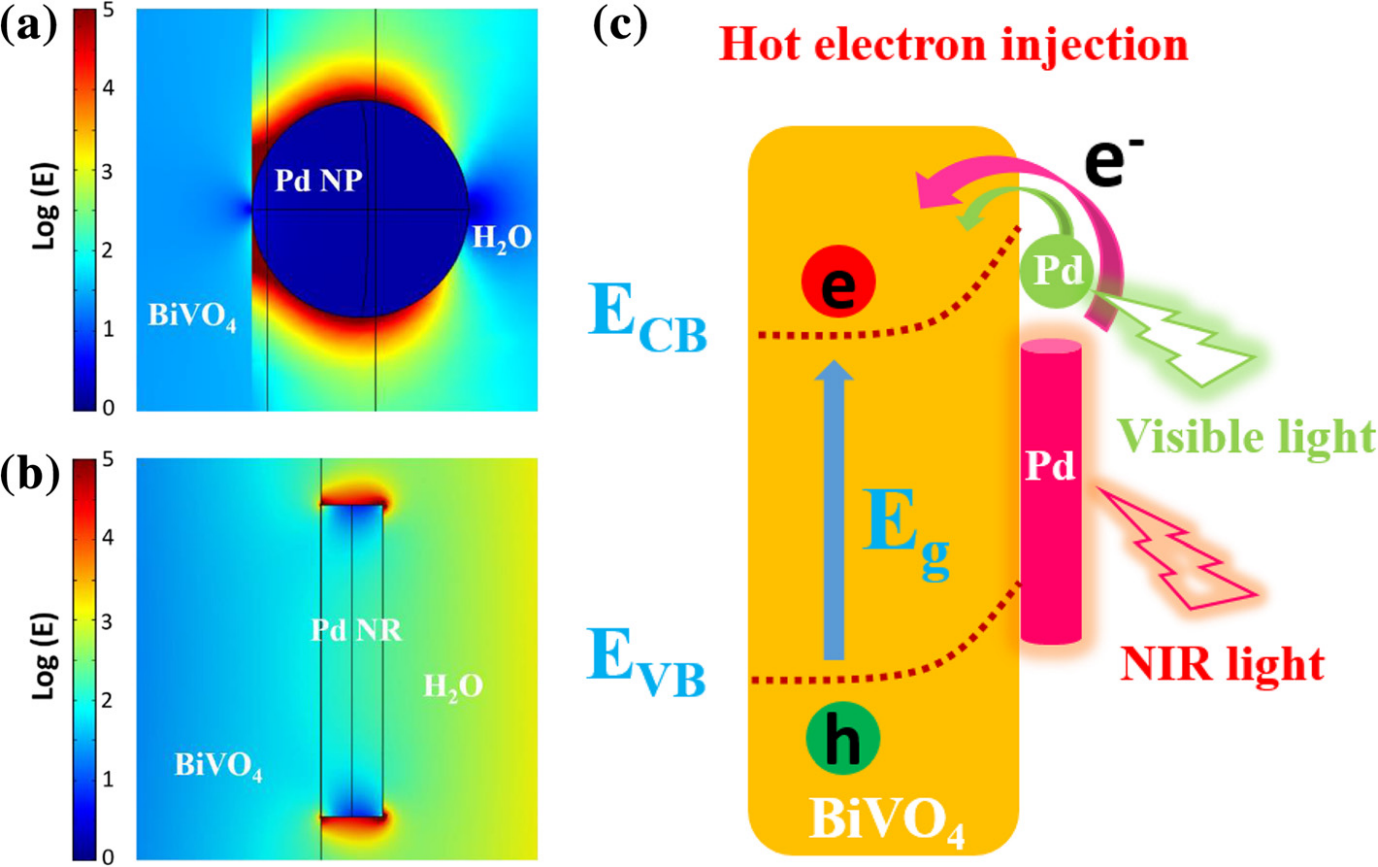
Spatial distribution of electric-field for **a** NP-BiVO_4_ and **b** NR-BiVO_4_ with excitation wavelengths of 500 and 900 nm, respectively. The incident light is along a specific direction (z axis). **c** Schematic illustration of the plasmon-induced charge carrier transfer under visible or NIR light illumination at Pd/BiVO_4_ interface

There are three energy transfer mechanisms by which SPR can enhance the concentration of charge carriers in a nearby semiconductor and therefore increase the photocurrent in a PEC cell. These mechanisms include resonant photon scattering, plasmon resonance energy transfer (PRET), and hot electron injection. First, the mechanism of resonant photon scattering can be ruled out for Pd nanostructure-decorated-BiVO_4_ electrodes containing Pd NPs with the diameter around 3.5 nm and Pd NRs with length and width of 11.8 and 1.3 nm, respectively, because it normally occurs in plasmonic metal nanostructures larger than 50 nm in size [34–36]. Second, the effect of PRET is normally observed at wavelengths where the plasmon resonance and semiconductor absorption overlap [37]. Whereas, the SPR feature of Pd NPs and NRs is located at 573 and 900 nm, respectively, indicating that there is barely no absorption overlap between Pd nanostructures and BiVO_4_ with an absorption band edge around 520 nm. Thus, the effect of PRET can also be ruled out for Pd nanostructure-decorated-BiVO_4_ electrodes. Third, the hot electron injection from plasmonic metal nanostructures into the conduction band of semiconductors is another possible process following the SPR excitation [38, 39]. Considering that the hot electrons accompany with an enhanced localized electric-field intensity in the vicinity of plasmonic metal nanostructures, the finite-difference time domain (FDTD) simulation was performed to calculate the spatial distribution of local electric-field intensity at the interface between Pd nanostructures and BiVO_4_ as a function of the wavelength of incident photons. Figure 7a, b shows typical FDTD simulation for Pd NP-BiVO_4_ and Pd NR-BiVO_4_ at excitation wavelengths of 500 and 900 nm, respectively. One can clearly observe that the electric-field intensity in the vicinity of Pd NP and Pd NR was greatly enhanced, indicating that there are substantial energetic hot electrons. Thus, in the present work, the hot electron injection mechanism could be the major contributor to the enhanced PEC performance of the Pd nanostructures decorated-BiVO_4_ electrodes in both visible and NIR regions. As schematically presented in Fig. 7c, under visible or NIR light illumination, Pd NP or NR acts as a sensitizer, absorbing resonant photons, generating the energetic hot electrons from the SPR excitation. Then, the hot electrons pass over the Schottky barrier at the Pd/BiVO_4_ interface and inject into the conduction band of BiVO_4_. Schottky barrier at the interface also helps the transferred hot electrons accumulate in the conduction band of BiVO_4_, preventing them from traveling back to the Pd nanostructures [32, 40].

## Conclusions

The photoactivity of Pd NPs and NRs decorated BiVO_4_ electrodes for PEC water splitting can be effectively enhanced across the visible-NIR region. The enhanced photoactivity in both visible and NIR regions was ascribed to the hot electron injection upon SPR excitation of Pd NPs and NRs, respectively. The present work aimed at enhancing the photoactivity of BiVO_4_ electrodes and at extending from the visible to the NIR region inspired us to design other plasmonic metal nanostructure-decorated semiconductor photoelectrodes for more effective utilization of the solar spectrum.

## Abbreviations

EDX, energy dispersive X-ray spectroscopy; FDTD, finite-difference time domain; LSVs, linear scanning voltammograms; NIR, near-infrared; NP-BiVO_4_, Pd NPs decorated BiVO_4_; NP-NR-BiVO_4_, Pd NPs and NRs decorated BiVO_4_; NPs, nanoparticles; NR-BiVO_4_, Pd NRs decorated BiVO_4_; NRs, nanorods; OECs, oxygen evolution catalysts; PEC, photoelectrochemical; PRET, plasmon resonance energy transfer; SEM, scanning electron microscopy; SPR, surface plasmon resonance; TEM, transmission electron microscopy; XRD, X-ray diffraction.
